# Supporting infants and parents in the perinatal period (SIPP): Co-creating an improved journey

**DOI:** 10.1371/journal.pone.0305786

**Published:** 2025-05-12

**Authors:** Tami Benzaken, Francesca Siracusa, Michelle D’Souza, Radhika Gulati, Esta Orchard, Alexandra Lemaigre, Clare Andrews, Mitch Blair

**Affiliations:** 1 River Island Academic Centre for Paediatrics and Child Health, Northwick Park Hospital, London North West University Healthcare NHS Trust, London, United Kingdom; 2 Imperial College School of Medicine, Faculty of Medicine, Imperial College London, London, United Kingdom; 3 Department of Primary Care and Public Health, Imperial College London, London, United Kingdom; University of Lausanne: Universite de Lausanne, SWITZERLAND

## Abstract

There has been a 70% increase in infant A&E attendance across England in the last decade, much of it non-urgent, highlighting the need to improve parental confidence and services for infant care. A multidisciplinary group of healthcare professionals was established with the aim of understanding and evaluating the support given to new parents in the early postnatal period. We recruited parents and caregivers of infants born in a large district hospital. Participants were recruited in the postnatal ward and neonatal unit. Participants were offered face-to-face or virtual interviews with an interpreter where required. Seventeen semi-structured interviews were conducted between February and September 2022 (16 virtual, 1 face-to-face). Thematic content analysis was used to manually identify codes and refined to develop a common coding framework which was used to identify final themes and subthemes. Following the initial set of interviews, a focus group with 12 different participants was held to validate the themes. Two main themes were identified: (1) service access, continuity, consistency, and personalisation of care are highly valued, and (2) preparation and support during transition is important and individual. Within theme 1, we identified facilitators (health literacy, consistent messaging, telephone line), barriers (staff shortages, communication between healthcare workers, discrimination), and person-centred care as sub-themes. In theme 2, participants highlighted the importance of their expectations of normal, support from family and friends, and experiences using applications and social media. Effective support and care in the postnatal period are vital for ensuring and promoting the health and wellbeing of mothers and their babies. These findings were presented to local maternity services and service users by the research team, resulting in local service improvements.

## Introduction

Despite improvements in child health with notable reductions in mortality and morbidity, children’s use of both primary and secondary healthcare in England has increased year-on-year over recent decades [1]. These trends are particularly apparent in emergency care settings, with infant emergency department visits increasing by almost 4% annually between 2007 and 2017 [2]. However, it is widely known from the literature that up to 40% of all attendances to paediatric emergency departments (EDs) are for non-urgent conditions [3]. Non-urgent attendances (NUAs) contribute to ED overcrowding, longer waiting times, and represent a growing challenge for healthcare systems [4–6].

NUAs are particularly common in infants, with children under 1 most likely to present to emergency services with a low-acuity issue [7]. In a retrospective study of infant attendance at London hospitals, 10% of infants had ‘nothing abnormal detected’ coded as a diagnosis. Presenting complaints for these infants included ‘unsettled baby’, ‘diarrhoea and/or vomiting’, ‘abnormal breathing’, and ‘feeding difficulties’ [8]. Jones *et al.* conducted a cross-sectional study of over 1.3 million infants in the UK and identified a 39% rise in hospital admission between 2008 and 2014 for infants with three diagnoses: physiological jaundice, feeding difficulties, and gastroenteritis [9]. These admissions were most prominent in neonates; 85% of the rise was contributed by infants 0–6 days old.

### What factors contribute to infant non-urgent attendance?

Why, when, and where parents seek healthcare is complex and multi-factorial. Pre-disposing factors for higher ED use include socioeconomic status (including immigrant and minority ethnic populations), language ability, health literacy, and income status [10,11]. Support networks for parents also play a crucial role – young or lone parents are more likely to present to ED [10] and frequent attenders to ED have less social support [12]. Caregivers often cite advantages of the emergency department, difficulty accessing primary care, perception of a serious/worsening condition, and need for reassurance as key reasons for NUA [6,13–15].

### Postnatal care service provision in England

Postnatal care in England is delivered primarily by midwives and health visitors (specialised community public health nurses). National Institute for Health and Care Excellence (NICE) guideline 194 outlines the postnatal care that women and babies should receive in the first 8 weeks after birth [16]. Prior to discharge from a maternity unit to community care, or prior to a midwife leaving after a home birth, an assessment of the woman and baby’s health should take place. This includes observing one effective feed. The first midwife visit after transfer of care to the community or after home birth should take place within 36 hours.

Routinely, a new mother will be discharged from midwifery care to health visiting between 10 and 14 days after birth. The first health visitor visit is often very soon after transfer of care, with the next at 6–8 weeks. This can leave a gap of several weeks between the first and second health visitor reviews; as a result, NICE guidelines suggest delaying the first home visit to 1–2 weeks after transfer of care to space visits out more evenly [16]. The postnatal check with a general practitioner (GP) should occur 6–8 weeks after birth.

Length of stay in hospital after both vaginal and caesarean birth in England has decreased over the last decade [17]; simultaneously, numbers of midwives and health visitors are dwindling [18]. Interprofessional working between midwives and health visitors (HV) is encouraged by the World Health Organisation, but restructuring of care, reduction in number of midwives and HVs, and disconnect with general practice means this is often not the case [19]. In a study of women’s experiences of maternity care in London, women identified a lack of continuity between the antenatal and postnatal period; they felt that communication between midwives and HVs was fragmented and limited, and they sometimes received conflicting advice [20].

### What does this study add?

Studies on infant NUAs provide valuable insight into parental decision-making and reasons for attendance, but this is only one snapshot in time and part of the picture. Our study seeks to understand more precisely what support parents report to receive in the immediate postnatal period, with a view to identify how services could better support them.

### Aims

#### Primary aim.

To understand what support is given to parents in hospital and the community in the first month after discharge from the postnatal ward and/ or neonatal unit.

#### Secondary aim.

To evaluate whether the support provided by the English NHS meets parents’ expressed needs and identify any other key support systems in the community.

## Methods

### Study population and recruitment

A combination of opportunistic and purposive sampling was used to recruit first-time parents and caregivers of infants born in Northwick Park Hospital between 5^th^ December 2021 and 30^th^ September 2022. Participants were recruited using a combination of individual approach and highly visible posters and leaflets in the postnatal ward, special care baby unit, and the neonatal unit with links to expression of interest forms to participate in the research.

Forty-two participants expressed interest in participation. Participants were re-contacted via telephone to organise an interview date with the aim of completing this within two months of their discharge date. Participants were given the option of a face-to-face or virtual interview via Microsoft Teams. Where English was not their first language, a virtual interpreter was organised. Prior to the interviews, parents were sent a participant information sheet and written consent was obtained.

### Interviews

Interviews were semi-structured using an interview guide (S1 Table). The guide was piloted within the research group and with parents not familiar with the research project. Prior to data collection, all researchers attended a virtual training session to familiarise themselves with the interview template by an experienced patient participation expert (EO). Interviews were conducted by female qualified doctors (MBBS) ranging in experience from Foundation Year 1 doctors to senior paediatric trainees. Interviewers had not previously met participants in clinical practice. No other individuals were present during interviews, except for a professional interpreter if needed. Interviews typically lasted between 30 and 45 minutes. All interviews were audio recorded and transcribed using Microsoft Teams. Transcripts were subsequently manually reviewed to ensure accuracy and were not returned to participants. Participants who had completed interviews were remunerated with a £20 e-voucher.

### Focus group

Following the initial set of interviews, a 90-minute focus group was held at a local ‘Baby Bank’. The focus group was run by a paediatric doctor (TB) and an expert in patient and public participation (EO). Twelve parents (11 mothers, 1 father) attended the workshop. Most parents had experienced the maternity services at Northwick Park Hospital. Anonymity of data was preserved as the parents were not the same as those who participated in the initial interviews so anonymised quotes could be used without causing discomfort to any individuals in the group. The focus group aim was to review and validate the themes that arose from the initial interviews and to explore potential future avenues for service improvement. During the session, parents discussed the interviews’ themes, their own experiences of postnatal care and recommendations to improve support available in the postnatal period.

### Data analysis

Thematic content analysis of interview transcripts was conducted to identify the support services used by parents, their experience of these, and the ways in which postnatal care could be improved [21]. Initial codes were manually derived inductively from two participant interviews. These preliminary codes were continuously reviewed and refined in subsequent interviews to develop a common coding framework by all authors. The coded data was reviewed to identify themes and subthemes.

### Ethical approval

Ethical approval was given by the London Northwest Hospital University Healthcare NHS Trust local ethics committee (service evaluation number: SE21/028).

## Results

Seventeen semi-structured interviews with new mothers were conducted between February and September 2022. Sixteen interviews were conducted using video calls and one face-to-face at Northwick Park Hospital due to participant preference. Participants were between 21 and 44 years old. Participants were ethnically diverse, representing our hospital local population: 8/17 reported Asian or Asian British ethnicity, 1/17 Black or Black British – African, 3/17 White British, 2/17 Other White ethnicity, 1/17 Mixed ethnicity, 1/17 Arab ethnicity, and 1/17 unknown.

We identified 2 major themes with 3 sub-themes pertaining to parents’ experiences and views of support received in the immediate postnatal period:

1. Service access, continuity, consistency, and personalisation are highly valued:
2. Facilitators: health literacy, telephone line, handover, consistent messaging, same carer, professional emotional support
3. Barriers: staffing shortages, shift work/handover, attitudes, inter-hospital and inter-professional communication, discrimination
4. Person-centeredness/personalised care
5. Preparation and support during transition is important and individual:
6. Expectations of “normal” and/or prior experience depends on health literacy:
7. Knowledge of health services and pathways
8. Specific subject knowledge: sleeping, feeding, bathing, maternal health
9. Practical and emotional support from family and friends
10. Roles of apps and social media, both positive and negative

### Theme 1: Service access, continuity, consistency, and personalisation are highly valued

Almost all mothers recognised the value of ease of service access, continuity of care, and personalisation of care. In the interviews, they raised several entities which were attributed as either facilitators or barriers to achieving such a service.

#### Theme 1a: Facilitators.

Several facilitating factors were broached by mothers. These included having practical and emotional support provided by healthcare professionals, consistency of staff, consistency of messaging, ensuring staff provide a clear and thorough handover between shifts, having access to telephone advice and being health literate.

A key facilitator raised by mothers was having access to practical support by healthcare professionals:

(Via interpreter) *‘But they (the midwife & health visitor) give advice on how to feed the baby. Also they give advice to give some vitamins. How to put the baby to sleep, to sleep in the cot because it’s safe for her and baby as well.’ (T32, 26-year-old, other White ethnicity mother, SCBU/NICU)*

*‘I was given support with breastfeeding from the first day and also told how to clean him, how to burp him, how to change his nappy, what sort of product that can be used on him. So a lot of things were hands-on.’ (T25, 31-year-old, Black or Black British – African, SCBU/NICU)*

This person noted the importance of not only practical support from healthcare professionals, but of emotional support as well.

*‘And then health visitor came... I really appreciated that she kept asking me about my mental health. That was good... and I did feel a bit more comfortable the second time because I knew there is some issue and I knew where to reach out and I knew we could all get help......I just called them today.’ (T41, 44-year-old, other White ethnicity mother, postnatal ward)*

In addition to support from professionals, parents valued support in terms of facilities available. This was particularly reported in the cohort of parents whose babies were admitted to the neonatal unit. They highlighted the importance of facilities to ‘room in’ and stay overnight with their baby on the unit prior to being discharged home.

*‘To have that facility was fantastic. I never knew something existed like that. The fact that me and my husband could stay in a room with baby and see how we get on.’ (T24, 36-year-old, Asian or Asian British - Indian mother, SCBU/NICU)*

Another facilitator to ensuring good service provision and supporting new parents was consistent messaging from healthcare professionals. This was raised by mothers as a facilitator, but more often highlighted as a barrier.

(Via interpreter) *‘Every single one gave the same advice to her. Then she can make a difference because everyone said exactly the same thing, which she had to follow for her health and for baby’s health and… it was very useful for her all of them.’ (T32, 26-year-old, other White ethnicity mother, SCBU/NICU)*

Finally, a key facilitator raised persistently by mothers was continuity of care, with regards to seeing the same healthcare professionals.

*‘Understanding the family, so continuity of care with someone that understands the family with the main point of contact.’ (T33, 33-year-old, White British mother, SCBU/NICU)*

*‘…the community midwife because she came here every week. She came here and was coming every week.’ (T36, 41-year-old, other White ethnicity mother, SCBU/NICU)*

#### Theme 1b: Barriers.

Mothers raised several barriers to ensuring good service access, provision, and support. These included system barriers such as staff shortages and shift working patterns (a major source of loss of continuity), as well as personal barriers related to individual healthcare professionals’ attitudes, communication, and, at times, even discrimination.

The inconsistency in healthcare providers, particularly midwives, was raised recurrently by mothers as a barrier to high-quality care and support.

*‘Ideally I would just like there to be one main point of contact just to make it easier and to make it less stressful. And I think that if you just had the main one point of contact, you would get to know each other better and you, you know, you get to understand each other and also to be able to spot the warning signals, like if the mental health isn’t quite there or you, you know, you’re struggling.’ (T33, 33-year-old, White British mother, SCBU/NICU)*

Situational barriers related to service infrastructure were highlighted by mothers. One recurrent barrier raised was the poor continuity of care due to shift work patterns, which would often result in inconsistent messaging and lack of familiarity of the patients and their situation.

*‘It kind of felt like we were going back to square one with every midwife because it was a new way of doing things or a slightly different advice, and which was really quite unhelpful.’ (T44, 34-year-old, White British mother, postnatal ward)*

*‘I’d have to be the one explaining what happened so far, and I just felt like I didn’t need to. It shouldn’t be coming from me.’ (T44, 34-year-old, White British mother, postnatal ward)*

Staff shortages were also flagged as a barrier for reduced access to services and poor service provision by mothers interviewed.

*‘The health visitor came to see only one time and then she never called back and that’s it.’ (T4, 29-year-old, unknown ethnicity mother, SCBU/NICU)*

*‘I can’t call the GP… no appointment available for next three weeks’ (T41, 44-year-old, other White ethnicity mother, postnatal ward)*

*‘Baby was rolled away straight away and I didn’t get to see him or anything, and no one really was telling me what was going on... But then not having porters or anyone to roll me upstairs meant I couldn’t see baby for, I think it was six hours that I didn’t see him for.’ (T26, 28-year-old, other Mixed ethnicity mother, SCBU/NICU)*

Poor communication from staff was highlighted as a cause of concern for parents with mothers reporting that they did not feel sufficiently updated. This was a particular concern when their babies were admitted to the neonatal unit.

*‘There wasn’t really any updates on what was happening with the baby.’ (T26, 28-year-old, other Mixed ethnicity mother, SCBU/NICU)*

*‘It was 11:00 o’clock at night and he didn’t know what was going on. And he just, yeah, rushed and came in and saw me and said I literally thought you were dying because no one said what was happening.’ (T26, 28-year-old, other Mixed ethnicity mother, SCBU/NICU)*

In addition to lack of communication, perceived attitudes from individual healthcare professionals towards parents were not always positive.

*‘I just felt a bit like the staff on that ward were a little bit dismissive.’ (T26, 28-year-old, other Mixed ethnicity mother, SCBU/NICU)*

*‘Some of them don’t want to answer or, you know, I felt that they’re rude to me. Then I just stayed quiet, because I’m staying there, I don’t want to say anything’. (T4, 29-year-old, unknown ethnicity mother, SCBU/NICU)*

Perceived discourteous communication was also reported by staff on the neonatal unit. Parents reported that they did not feel empowered to contribute to their babies’ care, but were also expected to do more.

*‘And I went there, and she said, excuse me, this is not a hotel where you can come and you can go. You need to stay with baby. I said I know, but the doctor told me that I can stay when I am free. But I also need to go back home and take my other child to nursery.’ (T36, 41-year-old, other White ethnicity mother, SCBU/NICU)*

The attitudes from a minority of healthcare professional were felt by mothers to be discriminatory at times. This was found to be particularly exhibited by midwives. The discrimination was felt to be related to multiple factors including maternal age, race and towards those who did not speak English as a first language.

*‘Every time I was reminded of my age. I just felt like I have been judged to have children this late.’ (T41, 44-year-old, other White ethnicity mother, postnatal ward)*

(Via interpreter) *‘Discrimination because we actually from other country we are immigrants.’ (T7, 32-year-old, Asian or Asian British – other ethnicity mother, postnatal ward)*

*(Via interpreter) ‘She says her husband speak English and they speak more with her husband.’ (T32, 26-year-old, other White ethnicity mother, SCBU/NICU)*

Finally, mothers highlighted the importance of having good health literacy and understanding of the structure of the healthcare system to feel well-supported in the postnatal period. Those who reported lower prior understanding of this and difficulty navigating the healthcare system found this to be a barrier to feeling supported.

(Via interpreter) ‘*It is difficult to know which exact number to call.’ (T9, 28-year-old, other ethnicity (Arab) mother, postnatal ward)*

*‘I was not aware that skin to skin is that important or keeping your baby next to you, because whenever somebody will come they will say, ‘Put the baby in the cot’.’ (T20, 26-year-old, Asian or Asian British, postnatal ward)*

#### Theme 1c: Personalised care.

Mothers highlighted how personalised care was key to ensuring they felt well-supported. This was a factor in both the physical care they received, but also with regards to patient records.

*‘On that ward, they took my care really seriously. I don’t know if it’s just ‘cause they could see how sick I was and I wasn’t able to keep anything down, but they were really helpful at keeping me updated or chasing doctors, getting prescriptions and making sure I got my medication, they were faultless.’ (T26, 28-year-old, other Mixed ethnicity mother, SCBU/NICU)*

*‘I think [we must] have a note on our files like because they know the situation that we’re in and they always seem to fit us in with like early morning appointments.’ (T33, 33-year-old, White British, SCBU/NICU)*

It was evident from the interviews that mothers felt that personalised care contributed to successfully achieving holistic care.

*‘It’s about talking to someone that you know understands the body and mind and everything.’ (T41, 44-year-old, other White ethnicity mother, postnatal ward)*

### Theme 2: Preparation and support during transition is important and individual

Analysis revealed the importance of both preparation and support during transitions for new mothers. This applied equally to the major transition of becoming a parent, and to more specific transitions such as transfer of location (e.g., different wards, discharge home) or transfer of care (e.g., midwifery to health visiting). Mothers highlighted that a good understanding of services and how to access them was imperative in ensuring a smooth transition.

Mothers expressed benefiting from support from a wide variety of sources, in particular healthcare staff and their partners. In all interviews, the importance of individualised care and advice was evident.

#### Theme 2a: Expectations of “normal”, and/or from prior experience and health literacy.

##### Part i) Knowledge of health services and pathways

As previously noted, a poor health literacy regarding services and pathways was highlighted as an important contributor to mothers not feeling well-supported during the postnatal period. Several mothers described not knowing who to contact if problems arose, and confusion around the roles of staff members they encountered.

(Via interpreter) *‘Make clear the [telephone] number and easier… to identify the reason for the appointment.’ (T9, 28-year-old, other ethnicity (Arab) mother, postnatal ward)*

(Via interpreter) *‘She is not sure who they are… I think they are the health visitors.’ (T9, 28-year-old, other ethnicity (Arab) mother, postnatal ward)*

Those with good prior knowledge of available healthcare services were able to access appropriate support through a variety of sources (e.g., GP, HV, pharmacist). However, there remained some reported difficulties with access to these services.

*‘But the main support, yeah, comes through GP. They’ve given me their numbers, I’ve got their personal numbers as well, some of them. So I’m always in link with them and if any problems, they told me to come in. I always know I’ve got the healthcare visitors [and] that I can go to the GP as well’ (T24, 36-year-old, Asian or Asian British – Indian mother, SCBU/NICU).*

*‘So I did call the health visiting team once just for some advice. It’s very, very hard to get hold of them. It took me over a week to get hold of someone and then a couple more days before… I could speak to someone.’ (T26, 28-year-old, other Mixed ethnicity mother, SCBU/NICU)*

(Via interpreter) *‘We asked the pharmacy that we think the baby has some cold and like stomach wasn’t good with a thing. And he gave some medication for baby. And after 1-2 days she felt better.’ (T9, 28-year-old, other ethnicity (Arab), postnatal ward).*

In one case, misunderstanding of health services was shown to be a barrier to a mother accessing appropriate support for her mental health.

*‘My sister told me too, ‘You can always go and ask for someone to talk, and then you can feel better.’ Then my mother-in-law said, ‘No, you cannot talk to anybody. If you’re gonna tell doctors or midwives, then they’re gonna take your child away, because they’re gonna say that you’re in depression, you can’t look after your child.’ (T4, 29-year-old, unknown ethnicity mother, SCBU/NICU)*

##### Part ii) Specific subject knowledge (e.g., sleeping, feeding, bathing, maternal health)

Mothers, even those with prior knowledge, consistently reported an appreciation for the information and support given to them by healthcare staff (midwifery, neonatal nurses, HVs) regarding specific aspects of how to care for their baby.

*‘He was quite sleepy when he was born, so he wouldn’t wake up to feed. It was quite worrying, but they were really, really good at alleviating my stresses.’ (T44, 34-year-old, White British mother, postnatal ward)*

*‘I think staying in the hospital that long also told me a lot of things to do with baby. Like if I hadn’t gotten all that advice and stuff. I don’t actually understand how people go straight home after giving birth. So all that advice and all that support is very important.’ (T25, 31-year-old, Black or Black British – African mother, SCBU/NICU)*

*‘I was given support with breastfeeding from the first day and also told how to clean him, how to burp him, how to change his nappy, what sort of product that can be used on him. So a lot of things were hands on.’ (T25, 31-year-old, Black or Black British – African mother, SCBU/NICU)*

*‘But because of what I was taught at neonatal, that’s why I knew exactly what to do’ (T25, 31-year-old, Black or Black British – African mother, SCBU/NICU)*

*‘The health visitor comes to my home and they go through every like information about baby vaccination, feeding and like clothing and everything.’ (T6, 24-year-old, Asian or Asian British – Indian mother, postnatal ward)*

#### Theme 2b: Partner and friends practical and emotional support.

Mothers consistently recognised the value of the support provided by their partners in the immediate postnatal period. Partners being allowed to stay with them at the hospital was highly valued. Of note, some COVID restrictions for visitors remained in place during the time that this cohort delivered their babies.

(Via interpreter) *‘My husband [is] really supporting me. And he knows that I’m supporting him’ (T4, 29-year-old, unknown ethnicity mother, SCBU/NICU)*

(Via interpreter) *‘Obviously she only had her husband support for the one day and he had to go home’ (T12, 25-year-old, Asian or Asian British – Bangladeshi mother, SCBU/NICU)*

*‘I cried, cried, cried even during the night. The whole night I cried. I called my husband, even the midwife, because my husband he couldn’t stay with me at hospital’ (T7, 32-year-old, Asian or Asian British – other ethnicity mother, postnatal ward)*

(Via interpreter) *‘It’s just her partner being there. He didn’t really have a chair. He didn’t really have anywhere to stay with her and it [was] not very comfortable for the partner’ (T12, 25-year-old, Asian or British Asian – Bangladeshi mother, SCBU/NICU)*

Support from a wider network of friends and family was also felt to be important by our cohort. The responses highlighted that new parents without such a network (for example those with families abroad) may feel more isolated following the birth of their child.

(Via interpreter) *‘The difference is having a baby in India there’s a lot of family support. Obviously, when she had the baby here, it’s only her and her husband’* (T39, 30-year-old, *Asian or Asian British mother*, SCBU/NICU)

*‘I have no family. My in-laws, they’re busy. My husband. But he’s been unwell at the moment. I have friends but they were working and all of my friends they don’t have kids.’ (T41, 44-year-old, other White ethnicity mother, postnatal ward)*

*‘If I didn’t have the other support around me and I have been relying on the health visitor, I’d be really stuck.’ (T33, 33-year-old, White British mother, SCBU/NICU)*

Several mothers also expressed that they felt partners would benefit from improved access to health education and support, to better be able to support the mothers and take on a caregiving role.

*‘Obviously there is certain stuff that I can only do as a mum, but I think that, you know, Dad should be more involved in the care of the baby. Particularly we want equality and life and we want to support women, particularly when we go back to work. And it made me realize that actually he has access to no healthcare professionals to help him with these. You know, these kind of questions and that doesn’t really seem very fair. And if he’s not able to ask these questions, I can imagine there’s quite a few fathers that if they don’t know how to interact with the child, they just won’t.’ (T33, 33-year-old, White British mother, SCBU/NICU)*

*‘A session as a family on like interacting with the baby ‘cause I wasn’t quite sure how to play with her, but like my husband the other day said to me, oh, I don’t know if I’m stimulating her enough’ (T33, 33-year-old, White British mother, SCBU/NICU)*

#### Theme 2c: Roles of apps and social media.

In general, most mothers interviewed stated a preference towards in-person teaching and peer support compared to information available online or through an app.

*‘You can go on YouTube all the time, but YouTube is not the same thing as having people there with you who are able to advise you and physically show you what to do.’ (T25, 31-year-old, Black or Black British – African mother, SCBU/NICU)*

*‘I can’t ask you through the video, ‘Oh, this, what I’ve done, is it okay? Is it alright?’ But in person you can, when you do something you can ask them to check… And they can show you, oh, you might need to fix this or do that. So in-person teaching is definitely much more effective than just watching something online’ (T25, 31-year-old, Black or Black British – African mother, SCBU/NICU)*

*‘I save up and ask … my WhatsApp group* [of other new parents met at the hospital]*, but you know they’re not the experts and we’re just kind of guessing amongst ourselves’ (T33, 33-year-old, White British mother, SCBU/NICU)*

For those that did use an app, there were some reported difficulties with ease of finding the required information and accessibility of the information for those whom English is not their first language.

*‘I don’t like it. I don’t think it’s very intuitive and I find it difficult to use, which is frustrating because there is lots of really good information on there when you can find it, but it feels very long winded’ (T33, 33-year-old, White British mother, SCBU/NICU)*

‘*As a new mum, I don’t have the time to be scrolling through to try and find the information that I’m looking for. I would like something where it’s easier to search*.’ *(T33, 33-year-old, White British mother, SCBU/NICU)*

(Via interpreter) *‘She’s not that good at online stuff at English, so I don’t think this will be helpful’ (T9, 28-year-old, other ethnicity (Arab) mother, postnatal ward)*

### Strengths and Limitations

This study was able to engage with a diverse group of participants representative of the local population. This was possible by utilising multiple sampling methods. Specifically, using a personal approach and interpreters in both participant recruitment and interviews helped reduce digital exclusion and ensured good representation of those with English as a second language. A further strength of this study is that a focus group took place within the local community, rather than at a healthcare facility. The focus group was held at a local Baby Bank where participants were part of the local community and many were familiar with each other. We believe that the prevailing sense of community enabled participants to feel safe, supported, and empowered, resulting in sincere and open responses during the focus group. The fact that there were trusted childcare arrangements at the venue certainly facilitated this.

Another key strength of this study was that the results were fed back directly to senior management at our hospital. As a result, service changes were made and embedded into local policy.

The study was predominantly carried out at a time where the UK was recovering from the COVID-19 pandemic. Consequently, many healthcare services, including maternity and child health services, were still affected by the pandemic. Participants were likely to have experienced reduced contact with healthcare professionals, limits on visitation, and higher than average vacancy rates amongst community midwifery services. These are likely to have limited participants’ experience, which is reflected in the results of the study. Furthermore, the study took place at a single site, and as such limits generalisability of the results.

The COREQ checklist was applied to this paper, ensuring transparency with regards to participant selection, setting, data collection, and analysis. Of note, all interviews were conducted by female healthcare professionals which may have elicited different information from participants, compared to if interviewers were male or not healthcare workers. Participants were explicitly told that information shared for research purposes would not impact their care. Participants were all given the opportunity to withdraw consent at any time. Funding for interpreters was available, minimising the impact of language barriers in those included. Themes were identified solely from data and not in advance of data collection.

## Discussion

Effective support and care in the postnatal period are vital for ensuring and promoting the health and wellbeing of mothers and their babies. Yet, in the wake of the recent Ockendon review, the question is raised as to why safety is still a concern in UK maternity services [22]. The Ockendon report highlighted 4 pillars for essential action: safe staffing, well-trained workforce, learning from incidents, and listening to families. This study focussed on the 4^th^ pillar: listening to families. The interviews conducted provided an insight into the experiences and perceived support women received in the immediate postnatal period and explored potential avenues for improvement.

Three predominant themes are worthy of further discussion: access to support, continuity of care and attitudes of healthcare professionals. Poor access to healthcare professionals in the immediate postnatal period was a recurrent theme raised by participants. This was largely attributed to reduced staffing. These included a range of staff including health visitors, midwives, GPs, and hospital porters. In the UK, the Health Visitor workforce has decreased by 37% since 2015 [23], the GP workforce continues to shrink with a 7.3% loss since 2015 [24] and the number of registered midwives continues to decrease year-on-year [25]. Moreover, participants expressed frustration at being unable to contact healthcare professionals when required. This is attributed in the wider literature as a contributory factor in the reduced uptake of routine postnatal care [26]. Parents were very much aware that staffing restrictions contributed to suboptimal patient-professional interactions.

Continuity of care for parents in the immediate postnatal period is highly valued. Participants highlighted the importance of practical and emotional support facilitated by good continuity of staff. Conversely, the lack of continuity and subsequent inconsistency in messaging were recognised as barriers in receiving good support by many. Almost all participants expressed a desire to be cared for by the same midwives throughout their pregnancy journey. Evidence has shown the benefit for both mothers and babies in the model for midwifery continuity of care [27,28].

The third key theme concerns the attitudes of healthcare professionals towards new parents. Participants appreciated staff showing concern and patience in their communication and valued clear and thorough explanations. However, at times participants reported that they felt they were not sufficiently updated. Moreover, a minority of participants reported discriminatory behaviour from staff towards them. Participants attributed this to being immigrants or English not being their first language. Sadly, we know that adverse pregnancy outcomes are attributable to socioeconomic and ethnic inequalities in England and so the perceived discrimination of patients within the service is of significant concern and must be addressed [29].

These findings have been included in a detailed report with several key recommendations presented to managers of local maternity services by the research team and one of the participants from the focus group, in the form of a face-to-face meeting. This resulted in a commitment being made for several changes including reinstating a maternity telephone advice hotline and cultural competency training for staff. In addition, suggestions were made by parents to improve experience which focused on staff behaviours such as awareness of facial expressions, need for considerable reassurance, regular checking/reviews, and improved handover communication. Parents were also acutely aware of limiting factors such as staff capacity and how this has a knock-on effect on such behaviours. This project also highlighted the need for improved data systems around perinatal emergency care and how reports from these can be used to better support families at risk.

## Conclusion

This study has successfully engaged parents from diverse backgrounds in helping us to understand and appreciate the lived experience of postnatal care in a large district hospital. In turn, the results have informed maternity service strategic developments in a spirit of co-production. In addition, the findings have been used successfully to bid for further funding to support integrated care systems at hyperlocal level with the aim of improving life course trajectories for parents and children living in the most disadvantaged parts of the city.

## Supporting Information

S1 File Interview Guide. (DOCX)
