# Supplementary material for: Supporting infants and parents in the perinatal period (SIPP): Co-creating an improved journey
Source: PLoS One. 2025 May 12;20(5):e0305786. doi: 10.1371/journal.pone.0305786 (PMC12068575; doi:10.1371/journal.pone.0305786)
Supplement: S1 File — (DOCX) [file pone.0305786.s001.docx]

**Supplementary Information**

**S1 Table: Semi-structured interview guide**

| **Introduction** | **Prompts** |
| --- | --- |
| Remind participant of aims & objectives  Intention to record, safety and anonymity of recordings  Who to speak to if any concerns  Confirm and record verbal consent |  |
| **Support** |  |
| Can you talk me through what support you received:  After your baby was born, whilst in hospital?  When you went home with your baby? | Prompt interviewee to start chronologically from birth of the baby/day 1 and talk through their experience.  Examples for prompting: midwife, health visitor, GP, other |
| Did you download the recommended “mum & baby app”? | Yes/No  If no – why not? |
| Have you downloaded any other apps (e.g. Baby Buddy, other)? | Yes/No  If yes – which one? |
| **Understanding Expressed Needs** |  |
| Was there anything you were expecting to find easy but found more difficult than expected? | Give examples if needed:  Issues regarding the baby: feeding, sleeping, growth/weight gain  Maternal/parental issues: mental health, physical health  Other: stress, finances, work, other children |
| Was there anything you were expecting to find difficult but found easier than expected? | Any of the above examples |
| **Assessing Impact of Support** |  |
| Of the support you received, which/who do you think was the most helpful and why? |  |
| Of the support you received, which/who was the least helpful and why? |  |
| Did you have a home visit? If yes, what was that like for you? | Midwife / home visitor / neo-natal nurse. |
| Have you been to a hospital/children’s centre/community clinic/GP for any routine check-ups? If so, what was that like for you? |  |
| **Quality Improvement** |  |
| If you could design the perfect support service for new-born babies, what would it look like? |  |
